# Core Self-Evaluations Affecting Retirement-Related Outcomes

**DOI:** 10.3390/ijerph18010174

**Published:** 2020-12-29

**Authors:** Sara Zaniboni, Gabriela Topa, Cristian Balducci

**Affiliations:** 1 Department of Psychology, University of Bologna, 47521 Cesena, Italy; 2 Department of Social and Organizational Psychology, National Distance Education University (UNED), 28040 Madrid, Spain; gtopa@psi.uned.es; 3 Department of Psychology, University of Bologna, 40127 Bologna, Italy; cristian.balducci3@unibo.it

**Keywords:** core self-evaluations, retirement preparation, retirement attitudes, retirement expectations, retirement goals

## Abstract

This study addressed a gap in the literature by examining the role of core self-evaluations as a predictor of retirement preparation (i.e., attitudes, expectations, and goals), compared to other important aspects such as demographic, financial, health, and work-related variables. Based on the resource-based dynamic model for retirement adjustment and the core self-evaluations theory, the present study showed that core self-evaluations significantly and positively affected the social component of retirement adjustment (H1), the retirement expectations of new beginning (H2), the retirement expectations of continuity (H3), and retirement goals (H4). Additionally, core-self evaluations negatively affected the retirement expectations of imposed disruption (H5). All the analyses were controlled for age, gender, perceived health, financial situation, job centrality, and expected retirement age. In conclusion, core self-evaluations are valuable and supportive to workers across the work lifespan, and for dealing with the retirement preparation.

## 1. Core Self-Evaluations Affecting Retirement-Related Outcomes

As the population ages, retirement adjustment and retirees’ wellbeing become topics of great concern for societies, policymakers, and especially for individuals [1]. An increasing number of empirical studies [2] and meta-analyses [3] suggest that retirees adjustment, as well as a wide range or retirement-related outcomes, can be affected by the retirement planning and preparation. Retirement has been defined as a process beginning with planning and preparing the retirement while workers are still at work [4,5]. In particular, an older worker starts to develop expectations, attitudes, intentions, and plans regarding retirement long before the actual end of the working life [6,7,8]. For example, a phase of remote anticipation has been set at the age of 50 to 55, when people start to manage their work and lives thinking about the future retirement [9,10]. Previous research underlined the importance of a deeper understanding of the retirement preparation phase [11]. One of the key reasons is that intervening in a phase that is quite far away from the effective retirement enlarges the margin of successful interventions, preventing negative outcomes later in the retirement. Indeed, a healthy retirement adjustment is important for a successful aging among older people [12], and it starts with an adequate preparation [13,14]. Research supports that retirement preparation fosters better adjustment to retirement [8,15]. For example, a meta-analysis by Topa and colleagues [5] showed a significant and positive relationship between retirement planning and retirement satisfaction. Therefore, understanding the key aspects that affect the retirement planning/preparation (i.e., expectations, attitudes, goals, plans) is important for helping older people to master the retirement transition and adjustment [12,14].

Previous studies have shown that different individual (e.g., age, expected retirement age, gender, health, financial situation, individual resources) and contextual aspects (e.g., job involvement/centrality, workplace age stereotypes/discrimination) can affect retirement planning/preparation [5,12,16]. However, psychological resources (e.g., self-esteem, self-efficacy, control, mastery, autonomy) have been less frequently studied compared to more material aspects (e.g., wealth), though they can greatly contribute to the retirement process, influencing how a person deals with the preparation-transition-adjustment phases [12,17,18]. In particular, core self-evaluations—defined as fundamental evaluations of an individual, regarding her/his own personal value, control, capacity, and stability [19]—have been scarcely investigated when studying the retirement process (i.e., planning/preparation). Specifically, only one cross-sectional study examined the effect of core self-evaluations on retirement-related aspects [13]. Muratore and Earl [13] found that core self-evaluations were positively related to retirement planning associated to self-protection. The self-protection domain includes personal non-financial preparations made by people to preserve wellbeing and health in later life (e.g., choosing a healthy lifestyle, engaging in social support networks, and searching for a safe and good physical environment) [16,20]. Despite the lack of empirical evidence on the relationships between core self-evaluations and retirement planning/preparation, different studies showed that core self-evaluations can have an important impact on work-related outcomes, such as wellbeing, performance, and work attitudes [21,22,23]. Therefore, research is needed to better understand the predictive role of core self-evaluations on retirement-related outcomes (e.g., planning/preparation). For example, insights into key individual aspects affecting the retirement preparation can improve the design of successful interventions that aim at preventing negative outcomes later in the retirement phase. 

The goal of the present study is to assess the role of core self-evaluations as a predictor of retirement-related outcomes (i.e., social adjustment, retirement goals and expectations), compared to demographic, financial, health, and work predictors. Specifically, we will assess whether core self-evaluations positively predict expectation of social adjustment, new beginning, continuity, and retirement goals on financial, health, and lifestyle aspects, and whether they negatively predict the expectations of imposed disruption. In the tested models, we will control for age, gender, health, financial situation, job centrality, and expected retirement age. Our work is based on the resource-based dynamic model for retirement adjustment [15] and the core self-evaluations theory [16].

### 1.1. Resource-Based Dynamic Model for Retirement Adjustment

As the nature of retirement changed, new theoretical models have been developed to better describe the phenomenon of exiting the labor market and, at the same time, how to maintain wealth, health, and personal wellbeing in later life [24]. 

The resource-based dynamic model for retirement adjustment [1,25] is an integrated theoretical approach that allows us to understand the individual differences in the retirement process, as well as the wide range of factors that can affect the retirement adjustment. The resource-based dynamic model, based on Hobfoll’s conservation of resources theory [26,27], proposes that retirement-related outcomes are affected by the individuals’ access to different kind of resources. Specifically, six domains of resources can affect retirement-related outcomes: physical (e.g., health), financial (e.g., salary and pension), social (e.g., social network), emotional (e.g., emotional stability), cognitive (e.g., working memory), and motivational resources (e.g., self-efficacy). People that have access to different resources can better satisfy their needs and they are less at risk of stress and anxiety potentially related to the retirement transition. On the other hand, resources scarcity—or even the threat of future limited resources—can lead to a negative adjustment, thus decreasing wellbeing during retirement. The resource perspective to the study of retirement process has received support [7,10,21]. However, most previous studies have been focused only on a restricted range of resources, such as health and wealth [4]. Indeed, there is still a lack of research on psychological resources compared to other resources such as material ones. For example, Hansson and colleagues [21] found that psychological resources (e.g., personal control) were more valuable than material ones (e.g., health and financial) for well-being during the retirement transition. More thorough studies on the role of psychological resources (i.e., like core self-evaluations) can contribute to a better understanding of the retirement process, since they are likely to affect how a person deals and get through the preparation-transition-adjustment. 

The present study will explore the effects of core self-evaluations (key personal characteristics/resources, see [16]) on retirement-related outcomes (e.g., social adjustment, retirement goals, and expectations). 

### 1.2. Core Self-Evaluations Theory

Core self-evaluations have been defined as fundamental evaluations of an individual regarding her/his own personal value, control, capacity, and stability [2,28]. Core self-evaluations are a second order personality construct that includes different features, which are self-esteem, generalized self-efficacy, internal locus of control, and emotional stability [22]. Individuals with positive core self-evaluations perceived themselves as stable, capable, and in control of their environment and their lives [19,29]. On the other hand, individuals with negative core self-evaluations perceived themselves as less valuable and capable than others, victims of their environment, and worried about their failures [23]. 

Due to the nature of core self-evaluations (i.e., self-evaluative focused, underling other more peripheral traits, and global/broad in scope), it has been argued that they are more strongly related to important work- and life -related outcomes, compared to other individual characteristics [16]. For example, a recent research showed that core self-evaluations significantly predicted important work and life outcomes beyond other individual characteristics, such as subjective age [30]. The authors suggested that, when it comes to work and life outcomes, it is more important how people feel about themselves in general (i.e., their core self-evaluations), than how old people perceive themselves.

Research has shown that core self-evaluations are related positively to team performance [31], task and organizational performance [32], servant leadership and leader effectiveness [33], life satisfaction, and emotional wellbeing [19]; on the other hand they are related negatively to work-family conflict [34], unemployment-related stress [35], perceived job insecurity [36], and withdrawal behavior [37]. Consistently with these findings, we can also expect that core self-evaluations can play a role in affecting retirement-related outcomes (e.g., expected social adjustment, retirement goals and expectations). However, except for one study [13], there is a paucity of research on the role of core self-evaluations in affecting retirement-related outcomes. Muratore and Earl [13] showed that core self-evaluations were positively related to planning effort in the self-protection domain (i.e., personal non-financial preparations finalized to preserve health and wellbeing in later life). However, the authors did not find significant effects of core self-evaluations in self-insurance (i.e., personal financial preparations that people do to optimize wealth in later life) or public protection planning (i.e., benefits given by the Government to promote health, wealth, and wellbeing in later life). Furthermore, Muratore and Earl [13] adopted a cross-sectional design, and assessed only a limited subset of aspects of retirement planning. Therefore, research is needed to better understand the role of core self-evaluations in the retirement process, since the extent to which people perceive themselves capable and in control of different situations can affect how they deal and cope with the retirement preparation-transition-adjustment.

### 1.3. Retirement Planning/Preparation, and the Role of Core Self-Evaluations

The retirement process starts with planning and preparing long before the actual leave of the workforce [17,32]. Retirement is a complex process, and it begins when people develop expectations, attitudes, and goals regarding their future life in retirement [17,38]. It is difficult to identify a precise age range in which people start to prepare the retirement. However, research on retirement preparation/planning frequently refers to a phase of anticipation around the age of 50–55, when people start to think and organize their lives considering their future retirement [5,10,11,12]. Previous scientific contributions on the preparation focused mostly on specific aspects of the decision process (e.g., intentions and behaviors related to prolong the working life, to have a bridge employment, to retire), and research is needed to better understand the complexity of this phase [39]. Retirement preparation and planning is not a unitary construct. Rather, it can be described as a set of attitudes (e.g., on social relations), expectations (e.g., of a new period of life), and goals (e.g., ongoing planning on different aspects such as financial, health, lifestyle, and psychosocial) related to different dimensions and spheres of the individual’s life [5,14,39,40].

Numerous evidence suggest that the preparation is important in achieving better retirement-related outcomes, such as adjustment, satisfaction, wellbeing e.g., [3,9,11,32]. Moreover, as suggested by the resource-based dynamic model for retirement adjustment [15,18] the access to important resources (e.g., psychological, physical, financial) can positively affect retirement-related outcomes (e.g., social adjustment, well-being). Therefore, the understanding of different aspects/resources (e.g., key personal characteristics such as core self-evaluations) that affect the retirement preparation/planning (e.g., expectations, attitudes, goals) is important for helping people to successfully retire [7,9]. 

Research has shown that different individual and contextual aspects, such as age and expected retirement age [8,39], gender [41,42], health [43], financial situation [44], job involvement/centrality [3,45], and psychological aspects/resources [7] can affect retirement planning/preparation. In particular, in term sof personal resources, some empirical studies showed that the first order factors (i.e., self-efficacy, self-esteem, internal locus of control, and emotional stability) included in the core self-evaluations significantly affected retirement-related outcomes. For example, both generalized self-efficacy and retirement self-efficacy can influence older people’ behaviors, such as retirement savings and retirement adjustment [46,47,48,49]. Self-esteem has been considered an antecedent of retirement adjustment and satisfaction [7,17,50]. Moreover, people with emotional stability and internal locus of control seem to be more active in planning the retirement [51,52]. Core self-evaluation theory [16,22] and related research results suggest that core self-evaluations are fundamental evaluations of an individual, and therefore more strongly associated to important work- and life-related outcomes compared to specific individual characteristics and aspects [24]. However, as previously mentioned, only one study examined the effect of core self-evaluations in relation to the retirement planning [13]. Considering the main limitations of the study (e.g., cross-sectional design and limited subset of aspects of the retirement planning), further investigations of the relationship between core self-evaluations and retirement planning/preparation are needed.

The present study, based on the resource-based dynamic model for retirement adjustment [15,18] and core self-evaluation theory [16,22], intends to assess the role of core self-evaluations as a key predictor of retirement-related outcomes (i.e., expected social adjustment, retirement goals and expectations). In particular, we expect that core self-evaluations would predict positively the social component of retirement adjustment (H1), the retirement expectations of new beginning (H2), the retirement expectations of continuity (H3), and retirement goals (H4), and would predict negatively the retirement expectations of imposed disruption (H5), over and above age, gender, perceived health, financial situation, job centrality, and expected retirement age.

## 2. Method

### 2.1. Participants and Procedure

The participants were 190 Spanish workers over age 50. The age of the participants was decided based on the age interval previously used by studies on pre-retirement phase [7]. The sample was 46.8% male (*n* = 89), and the average age was 55.3 years (*SD* = 4.36; range: 50–68). In addition, 50.6% (*n* = 96) had completed high school, 17.9% (*n* = 34) had some college education, 18.4% (*n* = 35) had professional training, and 13.2% (*n* = 25) attended graduate school. Regarding the job type, 54.2% (*n* = 103) of participants were technical workers such as machinists, sellers, and office workers, 20.5% (*n* = 39) were middle managers, and 14.7% (*n* = 28) were managers. Related to the occupational field, 31.6% (*n* = 60) of the respondents worked in the educational/health sector, 17.9% (*n* = 34) in factories, 16.8% (*n* = 32) in sales, 15.3% (*n* = 29) in police/military forces, 14.7% (*n* = 28) in banking and finances, and 3.7% (*n* = 7) in telecommunications. The average organizational tenure was 24.7 years (*SD* = 10.4). 

Graduate students helped in the data collection of the present research in the period between October 2019 and December 2019. They used their networks and social media to target a sample of people over 50, working in different sectors and jobs, that voluntarily accepted to participate in the surveys. Similar sampling strategies have been used in previous research works [53]. Data were collected online, through Google Forms, at two time points and surveys were paired via a code chosen by participants to maintain anonymity. At Time 1, participants provided information on demographic characteristics (i.e., age, gender, educational level, type of job, etc.), perceived health, financial situation related to family and retirement, job centrality, expected retirement age, and core self-evaluations. At Time 2 (2–3 weeks later), they provided information about social components of retirement adjustment, retirement expectations (i.e., new beginning, continuity, and imposed disruption), and retirement goals. The study received UNED Ethics Committee approval on 9 September 2019. The data that support the findings of this study are openly available in figshare at http://doi.org/10.6084/m9.figshare.12017898.

### 2.2. Analytical Approach

Preliminary examinations of the data were carried out. 

We tested for potential differences according to gender considering all the variables of the study. We did not find statistically significant differences between males and females regarding the main variables of our model (i.e., core self-evaluations, social components of retirement adjustment, retirement expectations of new beginning, continuity, imposed disruption, and retirement goals) and the control variables (i.e., age, health, financial situation, job centrality, and expected retirement age). Moreover, we did not find statistically significant differences between males and females regarding the demographic variables (i.e., education, job type, and occupational field) except for organizational tenure, t(188) = 2.59, *p* = 0.01, female (M = 22.85, SD = 10.35), male (M = 26.71, SD = 10.09).

The D’Agostino–Pearson test [54] was performed to test the normal distribution of the main variables of this study. The test was not statistically significant for core self-evaluations, retirement expectations of continuity, and retirement goals, while it was statistically significant (*p* > 0.05) for social component of retirement adjustment, retirement expectations of a new beginning, and imposed disruption. Considering the characteristics of this study (e.g., sample size, procedure of data collection, type of variables analyzed) and that the adopted analytical strategy (see below) tolerates moderate departures from the normality assumption [55], we proceeded in testing the study hypotheses.

The hypotheses were tested using Hierarchical Linear Regression Analysis with IBM SPSS [56]. In the first step of the analysis, we entered all the control variables (i.e., age, gender, health, financial situation, job centrality, and expected retirement age), while in the second step we included the focused independent variable (i.e., core self-evaluations).

## 3. Measures

### 3.1. T1 Core Self-Evaluations 

We used the validated Spanish version [57] of the 12-item measure designed by Judge and colleagues [58]. The scale assesses self-esteem, self-efficacy, emotional stability, and locus of control, yet, some items were not defined with the intention of being pure indicators of each of the four traits (i.e., some items may reflect the combination of two or more traits) [58]. A sample item is “I am confident I get the success I deserve in life.” Items are on a 5-point Likert scale ranging from 1 (*strongly disagree*) to 5 (*strongly agree*). The one-factor structure had high convergent and discriminant validity [51], and it has been confirmed in other validation studies of the scale in different languages [50,52,53]. The coefficient alpha in the present study was 0.71. 

### 3.2. T2 Social Component of Retirement Adjustment 

Twenty-three items from Fletcher and Hansson [54] were used to assess the attitudes about retirement, such as creating new social relations, adjusting the loss of social connections at work, and counting on family and colleagues support. A sample item is “Retirement will not bother me because I am sure I can make new friends no matter where I go.” Items are on a 5-point Likert scale ranging from 1 (*strongly disagree*) to 5 (*strongly agree*). The coefficient alpha in the present study was 0.93.

### 3.3. T2 Retirement Expectations of New Beginning, Continuity, and Imposed Disruption 

We used eleven items from the Retirement Expectation Inventory [55] rated on a 5-point Likert scale ranging from 1 (*strongly disagree*) to 5 (*strongly agree*). Three items assessed *New beginning* (i.e., retirement is the beginning of a new phase of life, with long-awaited goals, and the possibility to live life to the fullest), with a sample item being “Retirement will be the welcome beginning of a new stage of my life” (α = 0.77). Four items assessed *Continuity* (i.e., retirement is not a major change, the basic pattern of life goes on, with more time for activities); a sample item is “My life after retirement will be very similar to my life now” (α = 0.76). Four items assessed *Imposed disruption* (i.e., retirement is expected as meaningless and frustrating, since job is irreplaceable), with a sample item being “Nothing will be able to replace work in my life” (α = 0.83).

### 3.4. T2 Retirement Goals 

We used the Spanish version [56] of the 4-item measure created by Noone and colleagues [34]. The scale assessed retirement goal clarity regarding financial, health, lifestyle, and psychosocial. A sample item is “I have specific goals for my long-term health.” Items are on a 5-point Likert scale ranging from 1 (*strongly disagree*) to 5 (*strongly agree*). The coefficient alpha in the present study was 0.87.

### 3.5. T1 Control Variables 

The participants’ age, gender, health, financial situation related to family and retirement, job centrality, and expected retirement age were used as control variables. *Age* was included in the analysis since self-selecting factors (i.e., illness and death) can affect the fact that older workers are still at work. *Gender* was used as a control variable, since previous research showed that it may have a differential effect on the retirement process e.g., [35,36]. Moreover, *health* and *wealth* are important aspects in affecting the retirement age and adjustment [4]. Perceived health was assessed with one item (i.e., In general, how would you define your physical health?), on a 5-point Likert scale ranging from 1 (*not good at all*) to 5 (*totally good*). The financial situation was assessed with two items (i.e., “How much your financial situation is adequate for your future retirement?” and “How much your financial situation is adequate for your family need?”) on 5-point Likert scales ranging from 1 (*not at all*) to 5 (*completely*). *Job centrality* was included in the analysis since the attachment that a person has with his/her job has been found to correlate negatively with retirement decision and adjustment [5,45,57,58]. The construct was assessed with a single item: “In general, how important is your work in your life?” on a 5-point Likert scale ranging from 1 (*not important*) to 5 (*very important*). *Expected retirement age* is related to different aspects of the retirement planning/preparation [57], and it was included in the model between the control variables.

## 4. Results

Means, standard deviations, intercorrelations, and alpha reliabilities of the variables are presented in Table 1. Core self-evaluations is positively correlated to perceived health (*r* = 0.48, *p* < 0.01), financial situation (*r* = 0.33, *p* < 0.01), and job centrality (*r* = 0.21, *p* < 0.01). Perceived health is positively correlated to financial situation (*r* = 0.34, *p* < 0.01) and job centrality (*r* = 0.23, *p* < 0.01).

Hierarchical multiple regression analysis was used to test our models. In the first step, all the control variables (i.e., age, gender, health, financial situation related to family and retirement, job centrality, and expected retirement age) were included, and in the second step the core self-evaluations were added. Table 2 reports the results of the models tested. 

According to Hypothesis 1, we expected that core self-evaluations would positively predict the social component of retirement adjustment over and above the control variables considered (i.e., age, gender, perceived health, financial situation, job centrality, and expected retirement age). In Step 1, perceived health (*β* = 0.18, *p* = 0.02) and financial situation (*β* = 0.18, *p* = 0.02) positively predicted the social component of retirement adjustment, while age (*β* = −0.14, *p* = 0.04) and job centrality (*β* = −0.25, *p* = 0.00) negatively predicted the same variable. In Step 2, core self-evaluations emerged as a positive and significant predictor of the social component of retirement adjustment (*β* = 0.34, *p* = 0.00), over and above the control variables. The results supported Hypothesis 1: the addition of the core self-evaluations on Step 2 significantly increased the *R^2^*, *F*(1.182) = 19.60, *p* = 0.00, Δ*R*^2^ = 0.08.

According to Hypothesis 2, we expected that core self-evaluations would positively predict the retirement expectations of new beginning, over and above the control variables considered. In Step 1, none of the control variables significantly predicted the retirement expectations of new beginning. In Step 2, core self-evaluations positively predicted the same criterion (*β* = 0.27, *p =* 0.00). The results supported Hypothesis 2, with the addition of the core self-evaluations on Step 2 significantly increasing the *R^2^*, *F*(1.182) = 10.28, *p* = 0.00, Δ*R*^2^ = 0.05.

According to Hypothesis 3, we expected that core self-evaluations would positively predict the retirement expectations of continuity, over and above the control variables considered. In Step 1, perceived health positively predicted the retirement expectations of continuity (*β* = 0.16, *p* = 0.04). In Step 2, core self-evaluations emerged as a positive predictor of the same criterion (*β* = 0.19, *p =* 0.02). Such results supported Hypothesis 3: the addition the core self-evaluations on Step 2 significantly increased the *R^2^*, *F*(1.182) = 4.95, *p* = 0.03, Δ*R*^2^ = 0.02.

According to Hypothesis 4, we expected that core self-evaluations would predict positively retirement goals, over and above the control variables considered. In Step 1, perceived health negatively predicted retirement goals (*β* = −0.17, *p* = 0.03). In Step 2, core self-evaluations positively predicted the same variable (*β* = 0.26, *p =* 0.00). The results supported Hypothesis 4, the addition of the core self-evaluations on Step 2 significantly increased the *R^2^*, *F*(1.182) = 10.26, *p* = 0.00, Δ*R*^2^ = 0.05.

According to Hypothesis 5, we expected that core self-evaluations would negatively predict retirement expectations of imposed disruption, over and above the control variables considered. In Step 1, age (*β* = 0.16, *p* = 0.02) and job centrality (*β* = 0.22, *p* = 0.00) positively predicted the target variable. On the other hand, financial situation negatively predicted the retirement expectations of imposed disruption (*β* = −0.19, *p* = 0.01). In Step 2, core self-evaluations negatively predicted the retirement expectations of imposed disruption (*β* = −0.21, *p =* 0.01), supporting Hypothesis 5. Also in this case the addition of the core self-evaluations on Step 2 significantly increased the *R^2^*, *F*(1.182) = 6.60, *p* = 0.01, Δ*R*^2^ = 0.03.

## 5. Discussion

The purpose of this study was to address a gap in the literature by examining the role of core self-evaluations as a predictor of retirement-related outcomes (i.e., social adjustment, retirement goals and expectations), compared to other important demographic, financial, health, and work predictors (i.e., age, gender, health, financial situation, job centrality, and expected retirement age). The resource-based dynamic model for retirement adjustment [15] and the core self-evaluations theory [16] were used as theoretical frameworks for the hypotheses. As postulated, it was found that core self-evaluations positively predicted the social component of retirement adjustment (e.g., attitudes on the creation of new social relations, the adjustment of the loss of social connections at work), the retirement expectations of new beginning (e.g., expectations regarding the beginning of a new phase of life), and continuity (e.g., expectations about the stability of the basic pattern of life), and retirement goals (e.g., goal clarity regarding financial, health, lifestyle, and psychosocial aspects). Moreover, the results showed that core self-evaluations negatively predicted expectations of imposed disruption (e.g., retirement is expected as meaningless and frustrating, since job is irreplaceable), over and above age, gender, perceived health, financial situation, job centrality, and expected retirement age. Specifically, how older people generally feel about themselves (e.g., they perceived themselves as stable, capable, and in control of their environment and their lives) is more important than other aspects/resources in affecting how they prepare their retirement (e.g., expecting good social relations and time for new activities and a certain stability in life, setting specific goals on different dimensions such as financial, health, lifestyle, and psychosocial). 

### 5.1. Contributions to Theory and Research

As suggested by the resource-based dynamic model for retirement adjustment [15], retirement-related outcomes are affected by the individuals’ access to key resources (i.e., health, financial, social, emotional, cognitive, and motivational). However, previous studies have been focused mainly on material resources, such as health and wealth, and research is lacking on psychological resources (e.g., core self-evaluations). Moreover, psychological resources seem to be more important than material resources in affecting well-being related aspects in the retirement process [21]. Therefore, this contribution tried to answer to the need of more in-depth research on the role of psychological resources (i.e., core self-evaluations) in studying retirement-related outcomes (i.e., preparation).

Moreover, as suggested by the core self-evaluations theory [16] and related research [24], core self-evaluations are more strongly related to important work- and life -related outcomes, compared to other individual characteristics [16]. However, except for a single cross-sectional study on some specific aspects of the retirement planning [13], there is a paucity of research on the role of core self-evaluations in affecting retirement-related outcomes. Our study further explored this research topic showing that core self-evaluations (i.e., key psychological resources) affect the retirement preparation (i.e., attitudes on social adjustment, retirement goals and expectations), over and above other resources/aspects (i.e., age, gender, perceived health, financial situation, job centrality, and expected retirement age). In particular, we found that people with positive core self-evaluations (i.e., that perceived themselves as stable, capable, and in control of their environment and their lives) successfully prepare their retirement: they have positive attitudes regarding social relationships/networks, expectations of a new beginning or a stable period, and they plan specific goals on different aspects.

Furthermore, research supports that retirement preparation is important, and it promotes a better adjustment [10,11]. However, most research on the planning/preparation mainly focused on specific aspects of the decision process (e.g., intentions and behaviors related to prolong the working life, to have a bridge employment, to retire). Therefore, in order to understand the complexity of the retirement preparation it is necessary a more thorough consideration of the different aspects/dimensions of this phase [34]. Our study contributes to this direction, studying a large range of retirement preparation aspects, including attitudes, expectations, and goals related to different dimensions and spheres of the individual’s life (e.g., social, lifestyle, health, financial).

### 5.2. Practical Implications

The findings of this research have implications for individuals, societies, and policy makers. On the one hand, pension system reforms, particularly in developed countries, postponed the mandatory retirement age, therefore, older workers need to retain their jobs and to retire later. On the other hand, in order to increase the sustainability of health care systems, actions are needed to boost successful aging for older people, such as supporting the retirement adjustment. For example, maladjustment to retirement has been found to decrease mental health e.g., [18]. Therefore, we need to understand how to help older people to successfully age at work and during retirement. An increasing amount of research is focusing on understating how to keep older worker healthy, engaged and productive at work. However, little research is focusing on how to promote and prepare a healthy and fruitful retirement for these people. This contribution is answering to this call. 

Retirement needs to be taken into consideration while people are still at work, since how they prepare this phase will affect their wellbeing, adaptation, and satisfaction later in life. Moreover, focusing on the pre-retirement phase gives the advantage of a prevention over a reaction action, since there is still time to intervene and improve what future outcomes will be later in life. Therefore, it is important for different stakeholders (e.g., retiree associations, societies, and policy makers) to understand key aspects (e.g., psychological) that affect the retirement planning/preparation (i.e., expectations, attitudes, goals, plans), to help older people to successfully master the retirement transition and adjustment. In particular, this contribution underlies the need of broadening the aspects that should be taken into consideration for adequately preparing the retirement. Psychological resources seem to be more important than other material aspects (e.g., financial assets), that are frequently the main or only focus of retirement preparation interventions. 

### 5.3. Limitations and Future Research

Although this study found important research results, it also has some limitations. First, the data have been collected at two time points, 2–3 weeks apart, to deal to some extent with the common method variance issue [58]. However, longitudinal studies should be preferred not only from a methodological perspective but also from a theoretical perspective, providing insights on the different phases of the retirement process, such as preparation, transition, and adjustment. Therefore, future research should collect data through the full retirement process, for a deeper comprehension of the dynamic of the aspects related to this phenomenon. Secondly, we used a convenience sample of over 50 Spanish workers, heterogeneous in terms of jobs and sectors. This likely allowed for greater variability in the core variables of the study, but at the same time limited the understanding of specific job-/sector-related aspects, such as different retirement plans and needs. Moreover, we collected data on workers from a single country (i.e., Spain), which may limit the generalizability of our results, since retirement can be affected by national regulations. Future research should repeat the study comparing workers from different jobs and sectors, and from different countries. Third, we did not explore interactions between different type of resources, such as psychological and social. Previous research showed that personal aspects and social resources can interact in affecting retirement related-outcomes e.g., [7,21]. Therefore, we suggest that future research considers potential interaction effects of different resources, such as core self-evaluations and age climate in the workplace.

## 6. Conclusions

In conclusion, this study contributes to the literature by addressing a gap in the research about the role of core self-evaluations as a predictor of the retirement preparation (i.e., attitudes, expectations, and goals). Specifically, we found that core self-evaluations positively predicted attitudes on social adjustment, expectations of new beginning and continuity, and retirement goals on financial, health, and lifestyle aspects. Additionally, core self-evaluations negatively predicted expectations of imposed disruption. The obtained results held over and above age, gender, health, financial situation, job centrality, and expected retirement age. Future research should focus on the role of different resources (e.g., psychological and social) affecting the retirement preparation. 

## Figures and Tables

**Table 1:** Means, standard deviations, and intercorrelations among study variables (*n* = 190).

Heading	*M*	*SD*	1	2	3	4	5	6	7	8	9	10	11	12
Age	55.29	4.36	-											
2. Gender	0.47	0.50	0.06	-										
3. Perceived health	3.76	0.83	0.01	0.02	-									
4. Financial situation	3.29	0.95	0.08	0.08	0.34 **	-								
5. Job centrality	3.65	0.98	0.01	0.06	0.23 **	0.11	-							
6. Expected retirement age	64.95	6.00	−0.04	0.06	0.00	−0.16 *	0.04	-						
7. Core self-evaluations	3.44	0.51	0.06	0.00	0.48 **	0.33 **	0.21 **	−0.10	(0.71)					
8. Social components of retirement adjustment	3.93	0.68	−0.13	−0.05	0.18 *	0.20 **	−0.19 **	−0.05	0.33 **	(0.93)				
9. Retirement expectations of new beginning	3.98	0.80	0.01	−0.07	0.06	0.08	−0.11	−0.12	0.23 **	0.55 **	(0.77)			
10. Retirement expectations of continuity	3.29	0.90	−0.06	0.00	0.15 *	−0.04	0.14	−0.04	0.20 **	0.20 **	0.12	(0.76)		
11. Retirement expectations of imposed disruption	1.72	0.83	0.15 *	−0.10	−0.09	−0.18 *	0.18 *	0.02	−0.19 **	−0.83 **	−0.50 **	−0.12	(0.83)	
12. Retirement goals	3.10	0.98	0.04	0.05	−0.14 *	0.10	−0.15 *	−0.17 *	0.15 *	0.26 **	0.45 **	0.05	−0.22 **	(0.87)

Note: Cronbach’s alpha in brackets on the diagonal. * *p* < 0.05; ** *p* < 0.01.

**Table 2:** Results of the hierarchical multiple regression analysis (*n* = 190).

Heading	Social Components of Retirement Adjustment										
Step/Variable	*F*	*R* ^2^	Δ*R*^2^	*B*	*SE*	*β*	*p*	*B*	*SE*	*β*	*p*
Step 1 (control variables)	4.89 **	0.14	0.14 **								
Age				−0.02	0.01	−0.14	0.04	−0.02	0.01	−0.15	0.02
Gender				−0.07	0.09	−0.05	0.48	−0.05	0.09	−0.04	0.56
Perceived health				0.15	0.06	0.18	0.02	0.03	0.06	0.04	0.59
Financial situation				0.13	0.05	0.18	0.02	0.09	0.05	0.12	0.10
Job centrality				−0.17	0.05	−0.25	0.00	−0.20	0.05	−0.28	0.00
Expected retirement age				−0.00	0.01	−0.01	0.85	0.00	0.01	0.01	0.84
Step 2	7.41 **	0.22	0.08 **								
Core self-evaluations								0.46	0.10	0.34 **	0.00
**Step/variable**	**Retirement expectations of new beginning**										
	***F***	***R*^2^**	**Δ*R*^2^**	***B***	***SE***	***β***	***p***	***B***	***SE***	***β***	***p***
Step 1 (control variables)	1.33	0.04	0.04								
Age				0.00	0.01	0.01	0.90	0.00	0.01	−0.00	0.99
Gender				−0.10	0.12	−0.06	0.39	−0.09	0.11	−0.05	0.44
Perceived health				0.07	0.08	0.07	0.35	−0.03	0.08	−0.03	0.71
Financial situation				0.05	0.07	0.06	0.48	0.01	0.07	0.01	0.91
Job centrality				−0.11	0.06	-0.13		0.13	0.06	−0.16	0.03
Expected retirement age				−0.01	0.01	−0.11	0.15	−0.01	0.01	−0.09	0.23
Step 2	2.66 *	0.09	0.05 *								
Core self-evaluations								0.42	0.13	0.27	0.00
**Step/variable**	**Retirement expectations of continuity**										
	***F***	***R*^2^**	**Δ*R*^2^**	***B***	***SE***	***β***	***p***	***B***	***SE***	***β***	***p***
Step 1 (control variables)	1.68	0.05	0.05								
Age				−0.01	0.01	−0.06	0.41	−0.01	0.01	−0.07	0.35
Gender				0.01	0.13	0.00	0.95	0.02	0.13	0.01	0.89
Perceived health				0.18	0.09	0.16	0.04	0.09	0.09	0.09	0.30
Financial situation				−0.11	0.07	−0.11	0.15	−0.14	0.07	−0.15	0.07
Job centrality				0.11	0.07	0.12	0.11	0.09	0.07	0.10	0.17
Expected retirement age				−0.01	0.01	−0.07	0.34	v0.01	0.01	−0.05	0.45
Step 2	2.18 *	0.08	0.02 *								
Core self-evaluations								0.33	0.15	0.19	0.03
**Step/variable**	**Retirement goals**										
	***F***	***R*^2^**	**Δ*R*^2^**	***B***	***SE***	***β***	***p***	***B***	***SE***	***β***	***p***
Step 1 (control variables)	2.79 *	0.08	0.08 *								
Age				0.01	0.02	0.02	0.75	0.00	0.02	0.01	0.85
Gender				0.11	0.14	0.06	0.42	0.13	0.14	0.06	0.35
Perceived health				−0.20	0.09	−0.17	0.03	−0.32	0.10	−0.27	0.00
Financial situation				0.15	0.08	0.14	0.06	0.10	0.08	0.10	0.21
Job centrality				−0.12	0.07	−0.12	0.09	−0.15	0.07	−0.15	0.04
Expected retirement age				−0.02	0.01	−0.14	0.05	−0.02	0.01	−0.12	0.09
Step 2	3.98 **	0.13	0.05 **								
Core self-evaluations								0.50	0.16	0.26	0.00
**Step/variable**	**Retirement expectations of imposed disruption**										
	***F***	***R*^2^**	**Δ*R*^2^**	***B***	***SE***	***β***	***p***	***B***	***SE***	***β***	***p***
Step 1 (control variables)	3.93 **	0.11	0.11 **								
Age				0.03	0.01	0.17	0.02	0.03	0.01	0.17	0.01
Gender				−0.017	0.12	−0.10	0.15	−0.18	0.11	−0.11	0.12
Perceived health				−0.07	0.08	−0.07	0.33	0.01	0.08	0.01	0.92
Financial situation				−0.16	0.07	−0.19	0.01	−0.13	0.07	−0.15	0.04
Job centrality				0.18	0.06	0.22	0.00	0.20	0.06	0.24	0.00
Expected retirement age				−0.00	0.01	−0.01	0.92	0.00	0.01	−0.02	0.74
Step 2	4.42 **	0.14	0.03 *								
Core self-evaluations								−0.34	0.13	−0.21	0.01

Note: * *p* < 0.05; ** *p* < 0.01.

## Data Availability

The data that support the findings of this study are openly available in figshare at http://doi.org/10.6084/m9.figshare.12017898.
